# Draft genome of the mountain pine beetle, *Dendroctonus ponderosae *Hopkins, a major forest pest

**DOI:** 10.1186/gb-2013-14-3-r27

**Published:** 2013-03-27

**Authors:** Christopher I Keeling, Macaire MS Yuen, Nancy Y Liao, T Roderick Docking, Simon K Chan, Greg A Taylor, Diana L Palmquist, Shaun D Jackman, Anh Nguyen, Maria Li, Hannah Henderson, Jasmine K Janes, Yongjun Zhao, Pawan Pandoh, Richard Moore, Felix AH Sperling, Dezene P W Huber, Inanc Birol, Steven JM Jones, Joerg Bohlmann

**Affiliations:** 1 Michael Smith Laboratories, University of British Columbia, 301-2185 East Mall, Vancouver, BC, Canada V6T 1A4; 2 Canada's Michael Smith Genome Sciences Centre, 570 W 7th Ave #100 Vancouver, BC, Canada V5Z 4S6; 3 Department of Biological Sciences, CW 405, Biological Sciences Bldg., University of Alberta, Edmonton, AB, Canada T6G 2E9; 4 Ecosystem Science and Management Program, University of Northern British Columbia, 3333 University Way, Prince George, BC, Canada V2N 4Z9; 5 Department of Medical Genetics, University of British Columbia, University of British Columbia, 4500 Oak St., Vancouver, BC, Canada V6H 3N1; 6 Department of Molecular Biology and Biochemistry, Simon Fraser University, 8888 University Drive, Burnaby, BC, Canada V5A 1S6

**Keywords:** Coleoptera, Curculionoidea, Scolytinae, bark beetles, conifer, cytochrome P450, glutathione S-transferase, plant cell wall-degrading enzymes, horizontal gene transfer, sex chromosomes

## Abstract

**Background:**

The mountain pine beetle, *Dendroctonus ponderosae *Hopkins, is the most serious insect pest of western North American pine forests. A recent outbreak destroyed more than 15 million hectares of pine forests, with major environmental effects on forest health, and economic effects on the forest industry. The outbreak has in part been driven by climate change, and will contribute to increased carbon emissions through decaying forests.

**Results:**

We developed a genome sequence resource for the mountain pine beetle to better understand the unique aspects of this insect's biology. A draft *de novo *genome sequence was assembled from paired-end, short-read sequences from an individual field-collected male pupa, and scaffolded using mate-paired, short-read genomic sequences from pooled field-collected pupae, paired-end short-insert whole-transcriptome shotgun sequencing reads of mRNA from adult beetle tissues, and paired-end Sanger EST sequences from various life stages. We describe the cytochrome P450, glutathione S-transferase, and plant cell wall-degrading enzyme gene families important to the survival of the mountain pine beetle in its harsh and nutrient-poor host environment, and examine genome-wide single-nucleotide polymorphism variation. A horizontally transferred bacterial sucrose-6-phosphate hydrolase was evident in the genome, and its tissue-specific transcription suggests a functional role for this beetle.

**Conclusions:**

Despite Coleoptera being the largest insect order with over 400,000 described species, including many agricultural and forest pest species, this is only the second genome sequence reported in Coleoptera, and will provide an important resource for the Curculionoidea and other insects.

## Background

The order Coleoptera (beetles) is the most species-rich order of insects, with over 400,000 described species [1], yet to date only one coleopteran genome sequence has been published, that of the red flour beetle (*Tribolium castaneum*, superfamily Tenebrionoidea), a pest of stored grain products [2]. The superfamily Curculionoidea (weevils) diverged from Tenebrionoidea 236 million years ago (Mya) [3], and contains over 60,000 described species, including many of the world's insect pest species. One such group of pest species are the bark beetles (subfamily Scolytinae), encompassing over 6,000 species in approximately 220 genera. Currently, one of the most destructive bark beetle species is the mountain pine beetle (MPB), *Dendroctonus ponderosae *Hopkins. MPB has had a long recorded history of major outbreaks in western North America, and attacks many pine species (*Pinus *spp.) [4]. The current MPB epidemic far exceeds the scope of any previously recorded bark beetle outbreak, with over 15 million hectares of pine forests, predominantly lodgepole pine (*Pinus contorta*), infested in British Columbia alone [4]. In recent years, MPB has been found east of the northern Canadian Rockies, which was previously thought to be an effective geographical barrier [5]. This range expansion includes infestation of pine species not previously encountered by MPB, particularly jack pine (*Pinus banksiana*) and its hybrid with lodgepole pine [6]. As the predominantly jack pine boreal forest extends to the Atlantic coast, the potential for this beetle to spread further eastward is of major ecological, environmental, and economic concern [4,7].

MPB is one of 16 species of *Dendroctonus *described in the New World, with habitats ranging from Nicaragua to arctic North America [8], and additional single species in both northern Europe and Asia. As members of the tribe Tomicini in the Scolytinae, *Dendroctonus *have ancient associations with conifers [9]. Most *Dendroctonus *species are capable of killing their conifer hosts, an ancestral ability of this genus [10], and several species are considered serious pests. MPB is present over a wide latitude range, has thus adapted to wide temperature ranges, and has a measurable spatial genetic structure [11]. The ability of MPB and *Dendroctonus *in general to inhabit a wide range of latitudes may foretell future range expansion with anticipated climate change [4,12], and may substantially affect future carbon cycles as beetle-killed trees decay or burn to release their stored carbon [7,13].

The success of MPB and many other bark beetle species in overcoming the defenses of their conifer host [14,15] and in colonizing the trees is due in part to their pheromone-mediated mass attack on individual trees [16]. Both male and female beetles produce aggregation pheromones that effectively initiate and modulate the mass attack. These compounds are identical or similar across most *Dendroctonus *species and some other Scolytinae [17]. Another factor in their success in killing trees originates from the symbiotic fungi that the beetles vector to new host trees. These fungi, such as the pathogenic blue stain ascomycete, *Grosmannia clavigera *(for which extensive genomic resources are available [18-21]) infiltrate the sapwood of the tree and effectively block water transport. Both the beetles and associated fungi probably contribute to detoxification and metabolism of defense chemicals in the host pine.

Although transcriptomic resources are available for Scolytinae [22-24], the genome sequence of MPB will provide a valuable new reference for further studies in this and related Coleoptera. A challenge with assembling the genome of non-model organisms such as MPB is the difficulty or impossibility of obtaining highly inbred individuals to reduce the heterozygosity. As the cost of sequencing continues to drop, genome sequencing will be feasible for many more organisms, many of which are not practical or amenable to extensive inbreeding. Thus, the assembly processes must be adapted to resolve the issues of greater heterozygosity for assembly of diploid genomes into haploid, or alternatively diploid, assemblies.

We report the draft *de novo *genome sequence of MPB, and describe several highlights including the presence of a horizontally transferred gene, the identification of genome sequence representing the sex chromosomes, genome-wide single-nucleotide polymorphism (SNP) variation and distribution, and gene families with roles in host colonization.

## Results and discussion

### Assembly

Owing to the univoltine lifecycle of MPB and the difficulty in rearing it through many generations in the laboratory, we chose to sequence wild-collected insects. The genome sequence was assembled from over 400× coverage of short-read paired end tag (PET) sequencing of genomic DNA from an individual, field-collected, male MPB pupa, and was scaffolded with over 300× coverage of short-read mate-paired end tag (MPET) sequencing of 6,600, 10,000, and 12,000 bp fragment sizes with genomic DNA from a pool of mixed-sex pupae (Table 1). In addition, scaffolds were merged when supported by scaffold-spanning Sanger expressed sequence tag (EST) and/or RNA sequencing (RNA-seq) data from various life stages and tissues [24].

**Table 1:** Data used in assemblies and scaffolding

Sequence type	Number of reads, millions	read length, bp	Total coverage, Gbp	Fragment length, bp	Sequence coverage
Male PET	387	76-114	41	630	200×
Male PET	480	100-114	50	590	243×

Female PET	328	150	49	425	227×

MPET	610	76-101	59	6600	290×
MPET	72	51	3.7	10000	18×
MPET	90	51	4.6	12000	22×
RNA-seq	257	51-76	13	170	NA
Paired end Sanger ESTs	0.18	750	0.135	1100	NA

EST, Expressed sequence tag, MPET, mate-paired end tag; NA, not applicable; seq, sequencing..

Examination of the assembly identified a fraction of sequences from a gammaproteobacterium most similar to *Acinetobacter*. The microbial community associated with *Dendroctonus *spp. is known to be diverse, and *Acinetobacter *spp. have previously been shown to associate with bark beetles [25-27]. However, we could not ascertain whether the bacterial sequences originated from a symbiotic association with the original MPB pupa sequenced, as *Acinetobacter *sequences were absent from sequences originating from the MPB pupae used for scaffolding and the female adult assembly, suggesting an environmental association rather than a symbiotic relationship. A total of 264 scaffolds (2.4 Mbp) that only contained gene models with best matches to *Acinetobacter *genes were removed from the male MPB assembly after comparing the assembly with the complete genome of *Acinetobacter lwoffii *[28] using BLASTn and BLASTx.

After removal of *Acinetobacter *sequences, the assembly resulted in 8,460 scaffolds greater than 1,000 bp in size, and an N50 of 580,960 bp (Table 2). The reconstructed genome size of 204 Mbp was comparable with the estimated genome size of 208 Mbp for MPB (Gregory *et al*., submitted paper), a value similar to the 204 Mbp for *T. castaneum *[2]. The G+C content of the MPB assembly was similar to that of *T. castaneum *(36% versus 33% G+C, respectively) [2]. We identified 13,088 gene models, of which 92% were supported by significant protein homology with the National Centre for Biotechnology Information (NCBI) nr database and we found that 96.4% of the ultra-conserved core eukaryotic genes [29] in the male assembly were complete, and 3.2% were partial (Table 2).

**Table 2:** Assembly statistics

	**Male number**	**Female number**
Number of contigs	59583	40744
N50 contigs, bp	7451	10101
Largest contig, bp	225798	158947
Number of scaffolds >1,000 bp	8460	6547
N50 scaffolds, bp^a^	597806	382123
Number of scaffolds >N50	76	136
Largest scaffold, bp^a^	3746698	6768731
Reconstruction, Mbp^a^	202	213
Gene models	13088	12873
Ultra-conserved core eukaryotic genes, complete/partial, %	96.4/3.2	98.4/1.6

^a^Excluding Ns.

### Sex chromosomes and shared synteny with the *T. castaneum *genome

**Figure 1:**
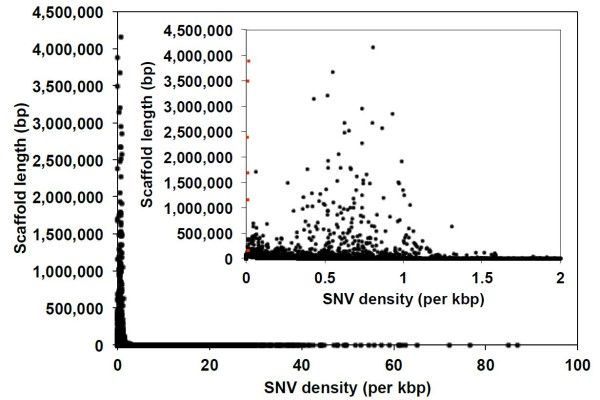
**Heterozygosity of an individual male**. The individual male mountain pine beetle (MPB) sequence data used for the assembly was mapped back onto the assembly, and the level of heterozygosity (allelic variation) was determined. Inset, A restricted range of single-nucleotide variant (SNV) density. Red markers indicate scaffolds with very low SNV density, which are hypothesized to represent scaffolds on the ancestral × chromosome portion of the neo-X chromosome. These are the six scaffolds shown in Figure 3.

Species in the genus *Dendroctonus *have a wide range of male meiotic karyotypic formulae, from 5 AA + Xy_p _in *Dendroctonus mexicanus *to 14 AA + Xy_p _in *Dendroctonus rufipennis *[30-33]. Identifying segments of the genome assembly representing the sex chromosomes in MPB has important implications for investigating sex-specific differences in MPB, such as pheromone production and size dimorphism, and how the sex-specific differences in this and other *Dendroctonus *species originate. In the case of MPB and its sibling species, *Dendroctonus jeffreyi*, the karyotype is 11 AA + neo-XY [31]. This arrangement (see Additional file 1, Figure S1) is thought to have originated from an ancestral state of 12 AA + Xy_p _by a fusion of the × chromosome with the largest autosome to become neo-X, followed by a loss of the ancestral y_p_, resulting in the homozygous daughter chromosome becoming neo-Y [32]. Therefore, the largest chromosomes in MPB are the neo-X and neo-Y sex chromosomes [31]. Given that we sequenced a male pupa, and males are the heterogametic sex, but physical or linkage maps are currently lacking, we attempted to identify scaffolds in the assembly that may have originated from the sex chromosomes. We hypothesized that the single-nucleotide variant (SNV) density should be very low for scaffolds that originate from the ancestral × portion of neo-X (as there would only be one copy in a male), or for regions of the neo-X and neo-Y originating from the ancestral autosome, which have sufficiently diverged to occur in the assembly as separate scaffolds (as there would be one copy of each in the male). To quantify this, we mapped the genomic DNA reads from the individual male back onto the assembly and measured the SNV density for each scaffold. We found that a few large scaffolds (Seq_1101913, Seq_1101939, Seq_1102308, Seq_1102689, Seq_1102713, and Seq_1102823) had very low SNV densities (as low as 0.006 SNVs per kb, for scaffolds greater than 1 Mbp) (Figure 1), whereas the overall SNV density of the assembly was 0.48 SNVs/kbp. There were smaller scaffolds up to 84 kbp with 0 SNVs/kbp, which may be additional unique pieces of the sex chromosomes.

**Figure 2:**
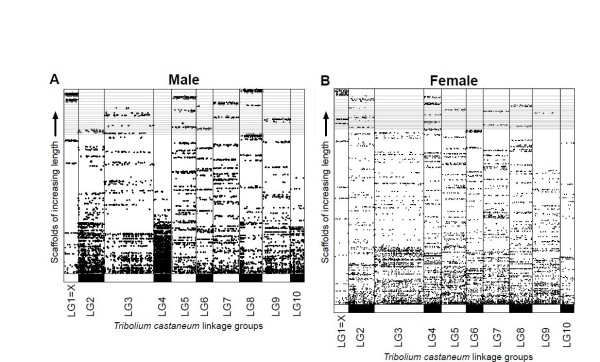
**Shared synteny between male and female mountain pine beetle (MPB) assembly scaffolds and *Tribolium castaneum *linkage groups**. Sequences were compared by tBLASTx and regions of significant similarity (e-value <1 × 10^-20^) are indicated by lines representing each high-scoring segment pair (appearing as dots at this scale). MPB scaffolds are displayed ordered from shortest to longest. The 20 longest MPB scaffolds are demarcated with faint horizontal lines. **(A) **Male and **(B) **female MPB scaffolds. (B) is longer than (A) due to a larger reconstruction size (including Ns) in the female compared with the male. A series of horizontal dots within one *T. castaneum *linkage group indicates a MPB scaffold sharing similarity with this linkage group. A linkage group for the y_p _chromosome in *T. castaneum *has not been described.

Although the common ancestor of MPB and *T. castaneum *diverged 236 Mya [3], we hypothesized that there may be conserved shared synteny between the two species, particularly in the × sex chromosome, even though male *T. castaneum *have a 9 AA+Xy_p _karyotype. We used tBLASTx to compare the MPB scaffolds with the linkage groups in the *T. castaneum *genome (Figure 2A). Consistent with our hypothesis, the six scaffolds identified with very low SNV densities matched strongly to LG1=X in *T. castaneum*, while the other long MPB scaffolds matched strongly to other *T. castaneum *linkage groups. We also found that scaffolds matching strongly to LG4 were noticeably shorter than scaffolds matching to other linkage groups. Owing to difficulties in assembling similar but divergent sequences shared between neo-Y and neo-X into a haploid assembly, partially redundant and shorter scaffolds were likely to result. This scenario would be consistent with LG4 sharing a common origin as the ancestral autosome that became neo-Y and part of neo-X in MPB. By contrast, sequence assembly from an individual female would not have these challenges (being neo-XX), so we sequenced an adult female beetle as well (Table 1, Table 2). Consistent with our hypothesis, we found that the scaffolds with gene models matching mostly to LG4 in *T. castaneum *(Figure 2B) were, on average, five times longer in the female MPB assembly than in the male MPB assembly, and the number of scaffolds was smaller (45 versus 98; Figure 2B). For the most part, the MPB scaffolds did not match defined positions in the *T. castaneum *linkage groups (Figure 2); rather, the matches were evenly spread out across a linkage group. This suggests that there have been significant intrachromosomal rearrangements since the separation of the two species from a common ancestor, more so than interchromosomal rearrangements. However, for scaffolds with gene models matching to linkage groups 3 and 8 in *T. castaneum*, some localization of the matches was apparent, possibly indicating past large interchromosomal rearrangements or fusions.

**Figure 3:**
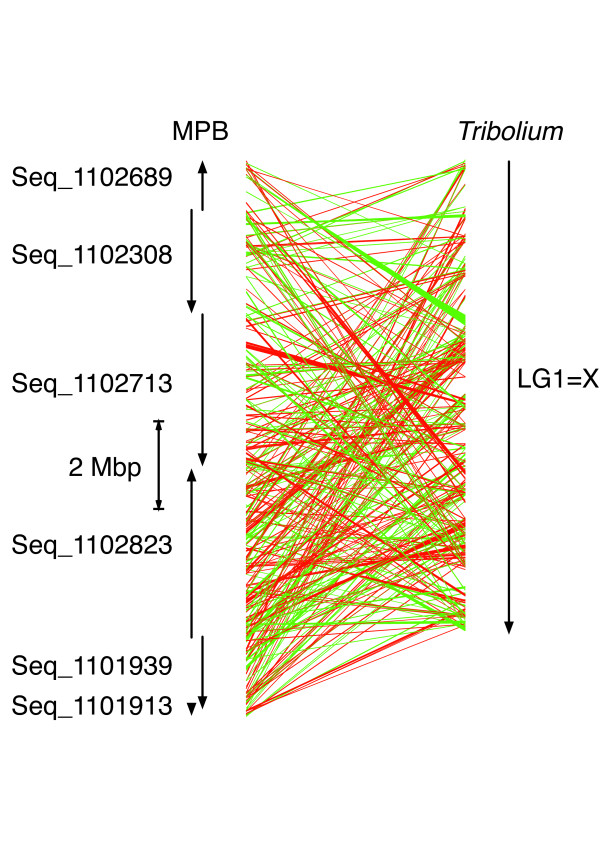
**Shared synteny between *Tribolium castaneum *LG1 = × and scaffolds representing the ancestral × portion of neo-X of the male mountain pine beetle (MPB) assembly**. Each trapezoid connects a matching gene model between the two organisms: red trapezoid, parallel orientation; green trapezoid, anti-parallel orientation. Scaffolds Seq_1101913, Seq_1101939, and Seq_1102823 partially overlap, and scaffolds Seq_1102689, Seq_1102308, and Seq_1102713 are contained in one scaffold in the female assembly. The order and orientation of these two groups of scaffolds are otherwise arbitrary.

To examine the shared synteny in more detail for LG1=X and the MPB scaffolds hypothesized to be part of the ancestral × portion of neo-X, we matched the gene models on these MPB scaffolds to the gene models in *T. castaneum *(Figure 3). Although approximately 70% of the gene models on these scaffolds matched gene models on LG1=X, large stretches of shared synteny were not apparent, indicating the extent of intrachromosomal arrangements that have occurred.

We thus identified scaffolds in the assembly that were likely to be on the sex chromosomes, but additional experimental data such as linkage maps are necessary for confirmation. In addition, we determined that although MPB and *T. castaneum *diverged more than 200 Mya, there was still evidence of shared synteny, although major intrachromosomal rearrangements were also apparent.

### Repetitive elements

Known arthropod and novel MPB repetitive elements were detected with RepeatMasker, RepBase Update, and RepeatScout. Repetitive elements occupied approximately 17 and 23% of the male and female genome assemblies, respectively (see Additional file 3, Table 1). This percentage is in the range of 8 to 42% that has been found in other insects [34]. Only 7% of the repetitive sequence had similarity to the known arthropod repeats in RepBase. The remainder appeared to be unique to MPB, with 3,429 and 2,941 novel elements appearing at least 10 times in the male and female genome assemblies, respectively. When these novel repeats were used to examine the *T. castaneum *genome, only 0.15% of this genome contained any of these novel MPB repeats, suggesting very little commonality between repeats in Coleoptera.

### Horizontal gene transfer

**Figure 4:**
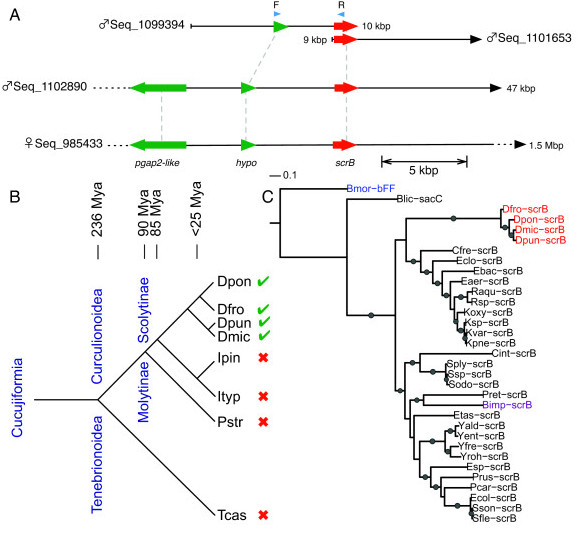
**Horizontal gene transfer**. **(A) **Schematic of the location of the sucrose-6-phosphate hydrolase gene (*scrB*, red arrow) on the male and female scaffolds. Green arrows indicate adjacent gene models with similarity to insect proteins by BLASTx (post-glycosylphosphatidylinositol (GPI) attachment to proteins factor 2-like (*pgap2-like*) and hypothetical protein, (*hypo*)). Blue arrows indicate the location of primers used to amplify intergenic regions between horizontal gene transfer (HGT) and adjacent beetle genes. Grey dashed lines indicate the similar gene models on the different scaffolds. **(B) **Presence of *scrB *in different beetle species. Green check marks and red Xs indicate presence and absence of the sucrose-6-phosphate hydrolase gene, respectively. Abbreviations: Dfro, *Dendroctonus frontalis*; Dmic, *Dendroctonus micans*; Dpon, *Dendroctonus ponderosae*; Dpun, *Dendroctonus punctatus*; Ipin, *Ips pini*; Ityp, *Ips typographus*; Pstr, *Pissodes strobi*; Tcas, *Tribolium castaneum*. Divergence dates estimated from Sequeira and Farrell [40]. **(C) **Phylogeny of scrB proteins from *Dendroctonus *(in red) with Gammaproteobacteria scrB and similar insect proteins. Abbreviations: bFF, beta-fructofuranosidase; Bimp, *Bombus impatiens*; Blic (in purple), *Bacillus licheniformis*; Bmor, *Bombyx mori*; Cfre, *Citrobacter freundii*; Cint, *Commensalibacter intestine*; Dfro, *D. frontalis*; Dmic, *D. micans*; Dpon, *D. ponderosae*; Dpun, *D. punctatus*; Eaer, *Enterobacter aerogenes*; Ebac, *Enterobacteriaceae bacterium *9_2_54FAA; Eclo, *Enterobacter cloacae*; Ecol, *Escherichia coli*; Esp, *Enterobacter *sp.; Etas, *Erwinia tasmaniensis*; Koxy, *Klebsiella oxytoca*; Kpne, *Klebsiella pneumoniae*; Ksp, *Klebsiella *sp.; Kvar, *Klebsiella variicola*; Pcar, *Pectobacterium carotovorum*; Pret, *Providencia rettgeri*; Prus, *P. rustigianii*; Raqu, *Rahnella aquatilis*; Rsp, *Rahnella *sp.; sacC, glycoside hydrolase; scrB, sucrose-6-phosphate hydrolase; Sfle, *Shigella flexneri*; Sodo, *Serratia odorifera*; Sply, *Serratia plymuthica*; Sson, *Shigella sonnei*; Ssp, *Serratia *sp.; Yald, *Yersinia aldovae*; Yent, *Yersinia enterocolitica*; Yfre, *Yersinia frederiksenii*; Yroh, *Yersinia rohdei*. Branches with dots had greater than 80% bootstrap support. The tree was rooted with *Bombyx mori *beta-fructofuranosidase (Bmor-bFF, in blue).

In the gene predictions of three male scaffolds and one female scaffold, we found a nearly identical gene, which had a high similarity match to sucrose-6-phosphate hydrolases (*scrB*) from enterobacteria, particularly *Klebsiella *spp. and *Rahnella aquatilis *(BLASTp e-value <1 × 10^-140^, 49% amino acid identity) (Figure 4A). These bacteria, especially *R. aquatilis*, are known to be associated with MPB [35], *Dendroctonus frontalis *[36], *Dendroctonus rhizophagus *[37], and *Dendroctonus valens *[27]. However, there was no evidence that other genes from these bacteria were present in the assembly. To confirm that this gene model was part of the MPB genome and not an assembly artifact of contaminating DNA, we successfully amplified and sequence verified a section of the genomic DNA about 4 kbp in length, which included both an adjacent beetle gene and the putative transferred bacterial gene. Transcripts corresponding to this locus (*Dpon-scrB*) are present in the transcriptome of beetles collected in different geographic regions in Canada [NCBI GAFW00000000 and GAFX00000000, 24] and the USA [NCBI dbEST GO486754, 23]. In addition, highly similar orthologous sequences (tBLASTn 88% identity, e-value <1 × 10^-165^) were found in transcriptome sequences from the southern pine beetle (*D, frontalis; Dfro-scrB*) (Keeling *et al*. in preparation; NCBI GAFI00000000). However, orthologous sequences could not be found in the available EST, nucleotide, and/or SRA transcriptomic data publically available at NCBI for more distantly related Scolytinae such as the coffee berry borer (*Hypothenemus hampei*, NCBI dbEST FD661949-FD663980), the pine engraver beetle (*Ips pini*) [22], or the European spruce beetle (*Ips typographus*) ([38], NCBI GACR01000000), or for other Coleoptera such as the white pine weevil (*Pissodes strobi; *NCBI GAEO00000000; Wytrykush *et al*, in preparation) and *T. castaneum*. We were also able to amplify the orthologous *scrB *using primers designed from the MPB and SPB *scrB *of the genomic DNA of the European great spruce beetle (*Dendroctonus micans*) and the North American Allegheny spruce beetle (*Dendroctonus. punctatus*) (Figure 4B,C). Amplicons from both of these two species shared 91% amino acid identity to the corresponding region of the MPB srcB, and were 97% identical to each other. These two spruce-infesting *Dendroctonus *species are closely related to each other, but are phylogenetically distant from MPB [39] and have different contemporary geographical ranges in Europe and North America, respectively. Although the depth of sequences available for other Scolytinae is limited and/or tissue-specific, the presence of this locus only in *Dendroctonus *species suggests that a horizontal bacterium-to-insect gene-transfer event may have occurred during or before the divergence of *Dendroctonus *species approximately 25 to 40 Mya [40,41], but after expansion of Scolytinae, approximately 85 Mya [3,41] (Figure 4B).

Most of the other gene models on the three scaffolds that contained *Dpon-scrB *matched to genes in *T. castaneum *on LG4. This evidence, and the presence of two loci in the male assembly and one in female assembly, suggested that this gene was located on the ancestral autosome portion of neo-X and neo-Y (Figure 4A). The closest match to an insect protein was a srcB-like protein from *Bombus impatiens *(XP_003494683, Bimp-scrB, Figure 4C), with 42% identity and an e-value of 5 × 10^-123^, which due to its closest neighbors being bacterial proteins (enterobacteria *Erwinia *spp., *Providencia *spp., and *Yersinia *spp.), may also be the result of a horizontal gene transfer, as has been described in *Bombyx mori *[42].

In bacteria, scrB catalyzes the hydrolysis of sucrose-6-phosphate to glucose-6-phosphate and fructose for carbohydrate utilization via the phosphotransferase system [43]. If this gene is expressed in MPB and is translated into a functional enzyme, it may contribute to the metabolism of carbohydrates in beetles. The Sanger EST sequences for this gene were obtained mainly from cDNA libraries originating from adult midgut/fat body tissue [24], while short-read RNA-seq data of separate adult midgut and fat body tissue (Keeling *et al*, in preparation) indicated expression in the midgut only. The specific expression of this horizontally acquired gene in digestive tissue suggests an adaptation of the beetle to facilitate digestion of host pine and/or fungal or bacterial carbohydrates. Based on the present analysis, in future work it should be possible to test such a function and role in *Dendroctonus *using biochemical or RNA interference methods. Another member of the Scolytinae has also recently been shown to harbor a horizontally transferred gene involved in carbohydrate metabolism. The coffee berry borer beetle (*H. hampei*) has a mannanase of apparent bacterial (*Bacillus*) origin [44]. An ortholog of this gene could not be found in either MPB assembly.

### Single-nucleotide polymorphisms

**Figure 5:**
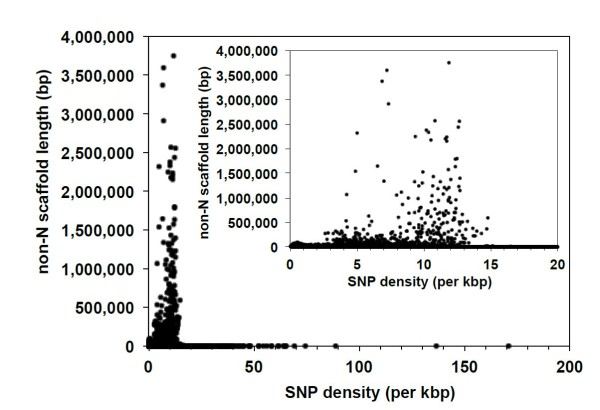
**Single-nucleotide polymorphism (SNP) density across the scaffolds for eight populations of beetles**. Inset shows a restricted range of SNP density.

To examine the variation and distribution of SNPs across the genome, we mapped short-read sequences of genomic DNA from pooled beetles (sampled from seven locations in Canada and one location in USA) to the male genome assembly, and then identified SNPs. We found a total of 1.69 million SNPs, with an allele that differed from the consensus sequence at a frequency of at least 6.25% in the sequence data from pooled populations. A small number of these, totaling 1.7% of all SNPs, were monomorphic relative to the consensus sequence. In addition, 97.8% of the SNPs were dimorphic, 0.5% were trimorphic, and 0.001% were tetramorphic. In total, 6.6% of the SNPs were found in exonic regions, 16.0% in intronic regions, and 77.4% in intergenic regions. These regions represent 8.4%, 13.0%, and 78.6% of the non-N portions of the assembled genome, respectively. On average, the SNP density was 7.55 SNPs/kbp (Figure 5) but varied between exonic (5.92 SNPs/kbp), intronic (9.27 SNPs/kbp), and intergenic (7.43 SNPs/kbp) regions. Although comparative data are limited in insects, MPB had a higher SNP density compared with the horned beetle (*Onthophagus taurus*) and the varroatosis mite (*Varroa destructor*), which average 5.67 SNPs/kbp [45] and 0.062 SNPs/kbp [46] respectively, but less than the 16.5 SNPs/kbp found in two *Lycaeides *butterfly species [47]. A comprehensive analysis of SNP variation across the geographic range of MPB and between the above eight populations is currently in progress.

### Orthology

**Figure 6:**
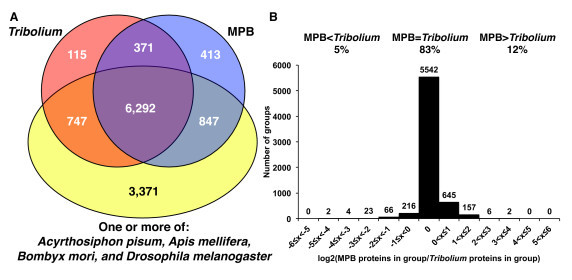
**Orthologs**. Comparative analysis of orthologous protein groups between six sequenced insect genomes. Predicted proteins from the genome sequences of mountain pine beetle (MPB), *Tribolium castaneum*, *Apis mellifera *(honey bee), *Bombyx mori *(silk moth), *Drosophila melanogaster*, and *Acyrthosiphon pisum *(pea aphid) were clustered into orthologous groups with OrthoMCL [84]. **(A) **Venn diagram indicates the number of protein groups found in either one or both beetle species among the 12,156 orthologous groups found. Numbers in parentheses indicate the percentage of these groups that were not found in any of the four non-beetle species. **(B) **Of the 6,663 groups found in both beetle species, 83% had an n to n correspondence between the two beetle species, whereas other groups had more or fewer members in mountain pine beetle (MPB) versus *T. castaneum*. The histogram indicates the distribution of the ratios of the number of MPB to *T. castaneum *proteins in each group.

The total number of 13,088 gene models identified in the male MPB assembly was similar to the number (16,404) of gene models initially found in the *T. castaneum *genome [2]. As this is only the second report of a beetle genome, we compared the protein predictions in MPB against those of *T. castaneum*, *Apis mellifera *(honey bee), *B. mori *(silk moth)*, Drosophila melanogaster*, and *Acyrthosiphon pisum *(pea aphid) by clustering them into orthologous groups to determine if there were coleopteran-specific orthologs, and whether the MPB genome has signatures of expanded or contracted gene families relative to *T. castaneum*. We found 413 protein groups, representing 1,055 predicted proteins, which were unique to MPB (Figure 6A). Of these predicted proteins, only 51 had no measureable expression based upon RNA-seq data from whole larvae and adult beetles (Keeling *et al*, in preparation). Of the 413 protein groups, 25% had no similarity to proteins in NCBI nr (e-value <1 × 10^-5^). The remaining 75% were similar to, but not deemed so similar as to be orthologous to, known proteins, and two-thirds of these were annotated to 'hypothetical proteins' in the other organisms.

There were 6,663 orthologous groups shared between MPB and *T. castaneum *(Figure 6A). Of these, most (83%) had an n to n relationship between the number of MPB to *T. castaneum *proteins in each group, but 12% of the groups contained more MPB proteins than *T. castaneum*, and 5% contained fewer MPB proteins than *T. castaneum *(Figure 6B). We found 371 groups that were present in both MPB and *T. castaneum *but not in any of the other compared species (Figure 6A), suggesting existence of some coleopteran-specific groups.

### Specific gene families

MPB spends most of its life cycle, except for a brief period of dispersal flight, under the bark of trees, where it is exposed to pine host defenses such as an abundance of oleoresin terpenoids [48]. Lignified cell walls and bark tissue that are rich in phenolics may also limit nutrients available for growth, development, and reproduction of the beetle. Thus, we anticipated that MPB has adapted to this hostile environment by evolving genes for detoxification of host pine chemicals and digestion of pine bark and wood tissue. We examined two gene families, the P450 cytochromes, and the glutathione S-transferases (GSTs), which are commonly involved in detoxification of plant chemicals, and from which some members are likely to be involved in the sequential pathway of metabolizing xenobiotics by making them more polar and excretable. Although the microorganisms associated with MPB are thought to facilitate the digestion of plant cell walls and lignins, and the concentration of nitrogen [49], insects have increasingly been shown to possess the ability to degrade plant cell walls themselves [50,51] and thus we also examined the family of plant cell wall-degrading enzymes (PCWDEs).

### Cytochrome P450 gene family

**Figure 7:**
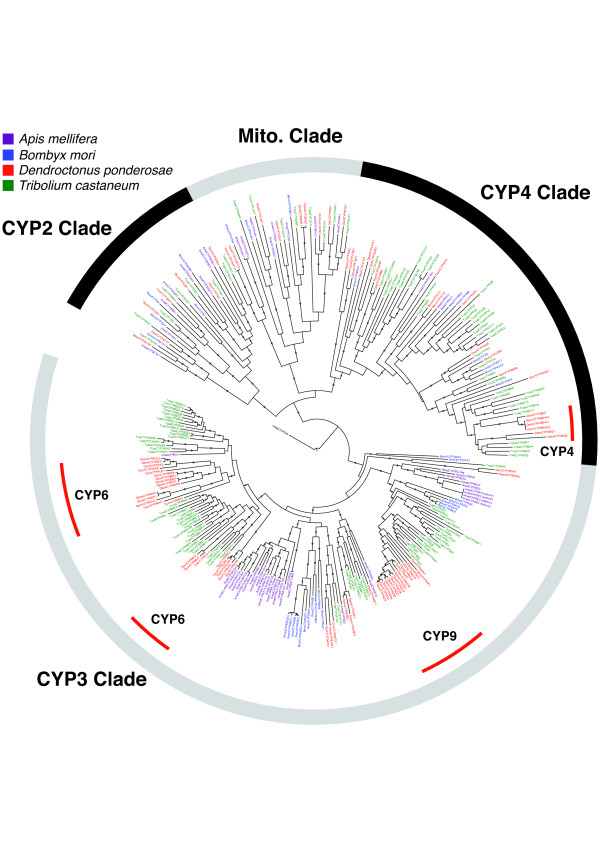
**Phylogeny of P450s**. Phylogeny of MPB (*Dendroctonus ponderosae*, Dpon) P450s with those from the honey bee (*Apis mellifera*, Amel), silk moth (*Bombyx mori*, Bmor), and red flour beetle (*Tribolium castaneum*, Tcas). Red arcs indicate areas of expansion of the mountain pine beetle (MPB) CYP4, and both CYP6 and CYP9 P450 families within the CYP4 and CYP3 clades, respectively. Branches with dots had greater than 80% bootstrap support. The tree was rooted with human (*Homo sapiens*, Hsap) CYP3A4.

The P450 cytochromes are a large family of enzymes associated with many processes of insect biology, particularly hormone biosynthesis, detoxification of xenobiotics (including plant defense compounds), pheromone biosynthesis, and insecticide resistance [52]. Insect P450s are found in four clades, CYP2, CYP3, CYP4, and mitochondrial, and are grouped further into families and subfamilies [52]. Members of CYP families share more than 40% amino acid identity (for example, the CYP9s), while members of subfamilies share more than 55% amino acid identity (for example, the CYP9Zs). For MPB, we found a total of 7 CYP2, 47 CYP3, 22 CYP4, and 9 mitochondrial P450s (Figure 7). This total of 85 P450s found in the MPB genome was less than the 134 identified in *T. castaneum *(8, 70, 44, and 9 in the respective clades), but it was within the range of the number of P450s found in other sequenced insect genomes [52,53].

We saw evidence for lineage-specific expansions ('blooms' [53]) in the CYP4, and particularly in the CYP6 and CYP9, MPB P450 families within the CYP4 and CYP3 clades, respectively (Figure 7). These blooms did not have orthologs in *T. castaneum*, nor did the blooms present in *T. castaneum *have orthologs in MPB. This pattern suggests that specific P450 family expansions have occurred in the lineages of each beetle species as part of their adaptations to different environments. The CYP2 and mitochondrial CYPs contained predominantly orthologous P450s across the species compared, and included the conserved P450s for 20-hydroxyecdysone biosynthesis (*DponCYP302A1*, *DponCYP306A1*, *DponCYP307A1*, *DponCYP307B1*, *DponCYP314A1*, *DponCYP315A1*) and deactivation (*DponCYP18A1*) [54], juvenile hormone biosynthesis (*DponCYP15A1*) [53], cuticle formation (*DponCYP301A1 *[55]), and circadian rhythm (*DponCYP49A1 *[56]).

We found several instances where several P450s were clustered on scaffolds, with nineteen clusters of two, five clusters of three, and one cluster of seven P450s. The cluster with seven P450s contained only CYP9Zs, and represented approximately one-half of all the CYP9s found in the MPB genome. In *T. castaneum*, five CYP9s were also found in a cluster. Very few CYP9s have been functionally characterized in any insect, but some can hydroxylate monoterpenes [57]. Only one P450, *DponCYP307A1 *(*spook*), was found on one of the putative ancestral × scaffolds (Seq_1102713). Its ortholog in *T. castaneum *is found on LG1=X.

Based on the observed patterns of specific P450 blooms in MPB, it is possible that these P450s have important functions for MPB to survive in the hostile chemical environment of the bark tissue of living pine trees. As genomes of other bark beetle species, including those that colonize dead trees or non-coniferous hosts, will be sequenced in future work, it should become possible to reconstruct the evolution of the observed blooms and to identify possible associations of such blooms with the colonization by MPB of living coniferous host trees, which are particularly rich in terpenoid and phenolic defenses. These diversified MPB P450 are also relevant targets for functional characterization, which is currently underway.

### Glutathione S-transferase gene family

GSTs are ubiquitous in organisms ranging from prokaryotes to animals. Although their functions are diverse [58], typical GST-mediated reactions involve the conjugation of glutathione with a substrate, often a toxin [59-62]. The addition of glutathione to the substrate increases the solubility of the substrate, aiding in detoxification and excretion. GSTs often act in concert with other enzymes, such as cytochromes P450 and epoxide hydrolases, which catalyze earlier reactions that prepare the substrate for the GST-catalyzed reaction [61].

Insects have six known classes of cytosolic GSTs (designated Delta, Epsilon, Omega, Sigma, Theta, and Zeta) plus a few individual entities that do not fit neatly into the current classification [63]. The Delta and Epsilon classes are unique to insects, are thought to be generally involved in detoxification reactions, and often contain a large number of representatives [58,61-63].

**Figure 8:**
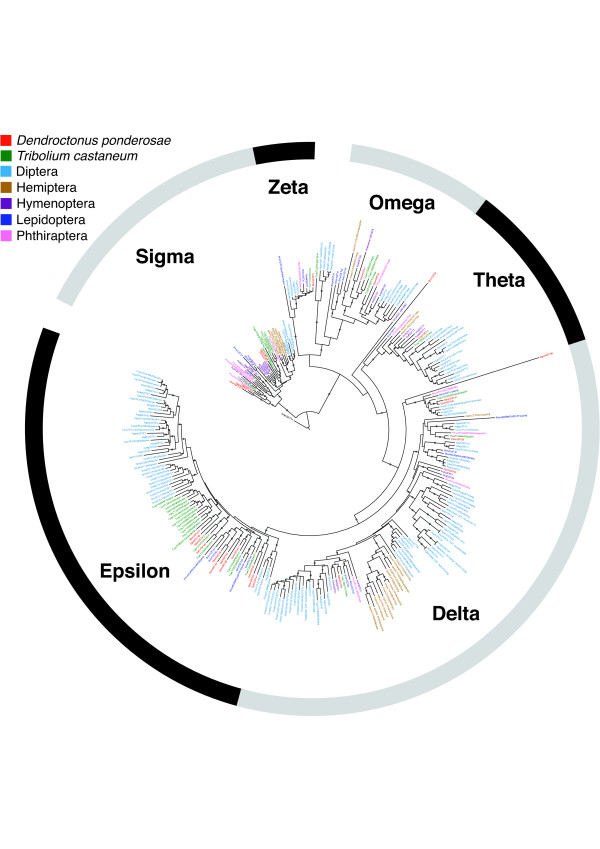
**Phylogeny of glutathione S-transferases (GSTs)**. Branches with dots had > 80% bootstrap support. The tree was rooted with human HsapGSTA1.Aaeg, *Aedes aegypti*; Agam, *Aedes gambiae*; Amel, *Apis mellifera*; Apis, *Acyrthosiphon pisum*; Bmor, *Bombyx mori*; Cqui, *Culex quinquefasciatus*; Dmel, *Drosophila melanogaster*; Dpon, *Dendroctonus ponderosae*; Hsap; *Homo sapiens*; Nvit, *Nasonia vitripennis*; Phum, *Pediculus humanus*; Tcas, *Tribolium castaneum*.

We found a total of 28 GSTs in the MPB genome (Figure 8), representing each of the six major classes. Specifically, we found 6 Delta GSTs, 12 Epsilon GSTs, 2 Omega GSTs, 5 Sigma GSTs, 2 Theta GST, and 1 Zeta GST. In many cases, the MPB GSTs had close orthologs in *T. castaneum*, which contains 3, 18, 3, 6, 1, and 1 GSTs, respectively, of the aforementioned families but obvious orthology was not always the case. The Epsilon class of MPB GSTs contained three small groups of genes (*DponGSTe1*, *DponGSTe2*, and *DponGSTe3*; *DponGSTe4 *and *DponGSTe5*; and *DponGSTe6 *and *DponGSTe7*) without orthologs in *T. castaneum*. Similarly, *DponGSTd3 *and *DponGSTd6 *did not have close *T. castaneum *orthologs. These GSTs without orthologs in *T. castaneum *may indicate an expansion of the GSTs in MPB or a contraction in *T. castaneum*.

As is a common phenomenon in gene families that arose from gene-duplication events, 16 of the 28 GSTs were found in clusters on genomic scaffolds. One small cluster contained two Sigma class GSTs (*DponGSTs1 *and *DponGSTs2*), while another cluster of two genes contained the only Zeta class GST found in MPB, along with one of the two Theta class GSTs (*DponGSTt1 *and *DponGSTz1*). The remaining two larger clusters contained all of the Epsilon GSTs; one contained five GSTs (*DponGSTe2*, *DponGSTe3*, *DponGSTe4*, *DponGSTe5*, and *DponGSTe8*), and the other contained the remaining seven GSTs (*DponGSTe1*, *DponGSTe6*, *DponGSTe7*, *DponGSTe9*, *DponGSTe10*, *DponGSTe11*, and *DponGSTe12*).

The 18 Epsilon and Delta GSTs identified in MPB indicate their likely importance in detoxifying the defense metabolite-infused pine tissue in which the larval and adult beetles exist. Whereas *T. castaneum *has had substantial exposure to pesticides in recent history, MPB has not. This may allow comparison of functions of orthologs between the two species, with the aim of understanding the development of GST-associated pesticide resistance. Some of the novel MPB GSTs, particularly from the Delta and Epsilon classes, will be investigated in future work for potential detoxification roles against secondary metabolites in pine.

### Plant cell wall-degrading enzymes

Plant cell walls are composed primarily of lignin and the carbohydrates cellulose, hemicellulose, and pectin. Microorganisms are effective at metabolizing these plant cell-wall carbohydrates for energy by using PCWDEs. MPB must be able to metabolize the cell walls of the woody pine species upon which it develops, either with the help of associated microorganisms, or on its own. Until recently, PCWDEs were thought to be absent in insects, because the sequenced model organisms such as *D. melanogaster *and *B. mori *lacked these genes. However, recent work has shown that PCWDEs are in fact both present and diverse in insects [50,51], particularly in the Coleoptera.

We manually annotated approximately 80 gene models coding for PCWDEs that were at least 100 amino acids long in the male genome assembly. These were reduced to 52 non-redundant PCWDEs by comparing protein translations including: six glycoside hydrolase family 48 proteins, seven polysaccharide lyase family 4 proteins, eight endo-b-1,4-glucanases, nine pectin methylesterases, and twenty-two endopolygalacturonases. Compared with a previous analysis [50] of our MPB Sanger EST data [24], we identified an additional nine PCWDEs in the MPB genome: two pectin methylesterases, two polysaccharide lyase family 4 proteins, and five endopolygalacturonases. In addition, we found three endopolygalacturonases that are probably pseudogenes. An ortholog to the *T. castaneum *glycoside hydrolase family 9 protein could not be found in either the MPB genome or transcriptome, although orthologs have been found in other insect species [51]. Although the identification of PCWDEs in insects has been limited, and has been studied most thoroughly in Coleoptera [50], MPB now has the largest family of PCWDEs described to date. In addition to understanding their roles in metabolizing plant cell walls for the nutrition of the beetle, and the relative role they play compared with that of the associated microorganisms in degrading the pine host tissues, there is also interest in the discovery of new insect PCWDEs for use in biotechnological applications, such as the degradation of cellulose for conversion of plant cell-wall sugars into biofuels or other bioproducts [64].

## Conclusions

Our demonstration of a successful *de novo *assembly of a draft genome sequence from short-read sequences from an individual insect without inbreeding to reduce heterozygosity is a forerunner to projects that aim to sequence non-model insects, such as in the i5k initiative [65]. The genome sequence of MPB provides a novel resource in Curculionoidea, many species of which are economically and ecologically important pests in forestry and agriculture. It also provides comparative data for the *T. castaneum *genome sequence to study the evolution of Coleoptera and insects in general. Our initial efforts to identify portions of the assembly corresponding to the sex chromosomes provide the framework for more comprehensive linkage studies using other approaches.

Our analyses of the MPB genome sequence identified unique features that may benefit the beetle for living in a plant defense-rich and nutrient-poor environment over a large geographic and bioclimatic range. The large number of SNPs identified here, when examined more thoroughly across multiple populations, will provide insights into the structure and natural variation in MPB populations throughout western North America, and may facilitate understanding of how the beetle is adapting to new hosts, new geographical ranges, and climatic conditions.

The presence of a bacterial sucrose-6-phosphate hydrolase in the genome sequence of MPB, which is also present in other *Dendroctonus *species with different hosts and different geographical ranges, and its tissue-specific expression in MPB midguts, suggests a unique functional integration of a horizontally transferred gene for carbohydrate utilization by MPB. Additional studies are necessary to confirm the role of this horizontally transferred gene in host colonization as a complement to the PCWDEs that were found in abundance in the MPB genome.

Some members of the P450 and GST gene families are probably involved in the detoxification of host defense compounds and in the production of pheromones that facilitate mass attack of the tree. The specific areas of expansion of these gene families relative to *T. castaneum *and other insect species hint at their roles in the specific biological processes necessary for MPB to be such a significant pest of pines.

## Methods

### Origin of beetles and DNA extraction

Pupae were chosen as the DNA source to minimize contaminating DNA from gut microorganisms and host tissue, because larvae void their gut prior to pupation. For PET sequencing, genomic DNA was extracted from an individual wild-collected male pupa removed from a bolt of a lodgepole pine (*P. contorta*) tree felled from along the Kay Kay Forest Service Road northwest of Prince George, BC, Canada (approx. N 54 02.731' W 123 19.109') in the fall of 2006. The sex of the individual pupa was established as male based upon microsatellite markers [66] on the isolated genomic DNA and the markers confirmed in the assembly. MPET sequencing for scaffolding used genomic DNA extracted and combined from 45 pupae, which were removed from one pine bolt obtained from a different tree, but felled at the same time and location. For sequencing the female genome, genomic DNA was extracted from an individual adult insect that emerged from a bolt collected at the same time and location as the pupae. Voucher specimens from the same cohort of insects have been submitted to the E H Strickland Entomological Museum at the University of Alberta and the Beaty Biodiversity Museum at the University of British Columbia. Frozen beetle tissue was homogenized using a cell disrupter (BeadBeater; Bio-Spec, Bartlesville, OK, USA) and the genomic DNA extracted (DNeasy Mini Plant Extraction Kit; Qiagen Inc., Valencia, CA, USA).

### Library construction and sequencing

For the male genome, two PET libraries with average fragment sizes of 590 and 630 bp were prepared with a commercial kit (Paired-End DNA Sample Prep Kit; Illumina Inc., San Diego, CA, USA) following the manufacturer's protocol (Paired-End Library Construction). For the female genome, one PET library with an average fragment size of 425 bp was prepared using the same protocol but a different kit (NEBNext Kit; New England Biolabs, Beverly, MA, USA). For scaffolding, three MPET libraries with average fragment sizes of 6,600, 10,000, and 12,000 bp were prepared (Mate Pair Library Prep Kit, version 2; Illumina) following the manufacturer's protocols but with some in-house modifications for the larger fragment sizes. The male sequencing and sequencing for scaffolding were completed on two different sequencing systems (GAII and HiSeq; both Illumina) and the female sequencing was completed on the HiSeq system.

### Assembly methods

#### Male assembly

The sequences generated from the two PET libraries for the male pupa and three MPET libraries for the pooled pupae were assembled using ABySS (version 1.3.0 [67]). The paired-end libraries were used for the de Bruijn graph assembly and paired-end assembly. The mate-pair libraries were used to scaffold the assembly, but were not used for their sequence data because of the chimeric reads produced by mate-pair libraries, and because the DNA originated from pooled beetles.

Mate-pair libraries contain a mixture of large-fragment reads that originate from DNA fragments containing the biotin label and of short-fragment reads that originate from DNA fragments that do not contain the biotin label. To enrich for large-fragment reads, we aligned the mate-pair reads to an earlier stage of the assembly, and removed read pairs that aligned with forward-reverse orientation rather than the expected reverse-forward orientation.

The ABySS assembly parameters were set to *k *= 64 for the de Bruijn graph assembly, *s *= 500 and *n *= 10 for the paired-end assembly, *s *= 1100 and *n *= 25 for scaffolding with the 6 kbp mate-pair library, and *s *= 3400 and *n *= 3 for scaffolding with the 10 kbp and 12 kbp mate-pair libraries. The parameter *k *is the size of a de Bruijn graph k-mer. The parameter *s *is the minimum size contig to consider placing in a scaffold, and the parameter *n *is the minimum number of paired read links required to merge two contigs in a scaffold.

Similar but not identical sequences of the genome are difficult to assemble, as they cause branches in the ABySS assembly graph referred to as bubbles. For a typical genome assembly, bubbles may be caused by near-repeats or by heterozygous variation. The MPB assembly faced these typical issues as well as the divergence of the neo-X and neo-Y chromosomes. The bubble-popping algorithm of the de Bruijn graph assembly stage of ABySS popped bubbles that were shorter than 3*k *in length, allowing for two SNV within *k *of each other. SNVs separated by more than *k *formed two separate bubbles, which were popped individually. After the de Bruijn graph assembly was performed, bubbles longer than 3*k *were popped if the two branches of the bubble had a minimum 90% identity. When a bubble was popped, the sequence with the most coverage was selected to represent the bubble. The other branch of the bubble was stored in an auxiliary file.

A sequence overlap graph represents each sequence as a vertex and each overlap of two sequences as a directed edge. For a simple bubble between two vertices *u *and *v*, the out-neighborhood of vertex *u*, N+(*u*), is identical to the in-neighborhood of vertex *v*, N-(*v*) (see Additional file 2, Figure 2A). For a complex bubble, all paths starting from vertex *u *must eventually pass through vertex *v*, but the structure of the subgraph inside the bubble is more complex than a simple bubble (see Additional file 2, Figure 2B).

ABySS identified complex bubbles when the bubble subgraph was a directed acyclic graph. Rather than pick a representative sequence or consensus sequence, ABySS scaffolded over the complex bubble by replacing it with a span of Ns in the assembly, whose length represented the longest path through the bubble. Both simple bubble-popping and scaffolding over complex bubbles increased the N50.

EST sequences [24] and contigs assembled by ABySS from RNA-seq data were aligned to the genomic scaffolds, and expressed sequences that spanned multiple scaffolds were used as evidence to merge those scaffolds, requiring a minimum of two expressed sequences from different libraries supporting the same merge. The two scaffolds in such merges were separated by a gap of 100 Ns. Finally, we used Anchor 0.2.7 [68] to correct small misassemblies, reduce or close scaffold gap sequences, and extend the ends of scaffold sequences. Only the paired-end libraries were used with Anchor.

#### Female assembly

The female assembly was generated in a similar manner to the male assembly, except that ABySS version 1.3.3 with k = 96 and Anchor version 0.3.1 were used. The same MPET sequences as for the male assembly were used for scaffolding.

### Annotation

The male MPB genome assembly was initially annotated using the MAKER [69] pipeline, limiting annotation to scaffolds over 1 kb in length. This software synthesizes the results from *ab initio *gene predictors with experimental gene evidence to produce final annotations. Within the MAKER framework, RepeatMasker [70] was used to mask low-complexity genomic sequence based on the RepBase Coleoptera repeat library [71]. Also within the MAKER framework, AUGUSTUS [72], Snap [73], and GeneMark [74] were run to produce *ab initio *gene predictions. AUGUSTUS predictions were based on the included *T. castaneum *training set of genes, whereas Snap gene predictions were based on its own minimal training set, and GeneMark was self-trained. These three sets of predictions were combined with the BLASTx [75], BLASTn [75], and exonerate [76] alignments of 178,536 clustered EST sequences [24], 12 RNA-seq libraries (Keeling *et al*, in preparation) assembled with ABySS, and protein sequences from *T. castaneum *[2] to produce the final annotations. Known protein domains were then further annotated using InterProScan [77]. Both assemblies were also compared with the ultra-conserved core eukaryotic gene dataset [29] to assess completeness.

### Analysis of sex chromosomes and shared synteny with the *T. castaneum *genome

Lacking linkage or physical maps, we attempted to identify portions of the sex chromosomes (especially the ancestral × portion of the neo-X chromosome) *in silico*. First, we mapped the genomic sequencing reads from the individual male pupa to the assembly with BWA [78] and then called SNVs with mpileup in SAMtools [79] with a minimum of 10-fold coverage. Scaffolds originating from the ancestral × portion of neo-X would be expected to have very low (theoretically zero, excluding sequencing errors) SNV density, as only one copy would be present in a male genome, whereas scaffolds originating from merged portions of the ancestral autosome portion of neo-X and neo-Y would be expected to have high SNV density because there are two copies in the male (from neo-X and from neo-Y). We hypothesized that the latter would be more divergent than autosomes because of reduced recombination, and that scaffolds originating from the ancestral × portion of neo-X would be longer on average because of more efficient assembly resulting from the presence of only one allele.

The level of shared synteny of the MPB assemblies to the linkage groups in *T. castaneum *was determined by tBLASTx (e-value <1 × 10^-20^), and the resulting HSPs drawn in R [80]. To compare the scaffolds of the putative ancestral × portion of the male MPB assembly with LG1 = × in *T. castaneum*, we used BLASTp (e-value <1 × 10^-25^) with the translated MPB gene models to identify the most similar translated *T. castaneum *gene models. We then determined the linkage group and position of the *T. castaneum *gene models using data from NCBI and BeetleBase [81], and plotted the matching pairs in OmniGraffle Pro (version 5.4) using a custom Applescript.

### Analysis of repetitive elements

Known repetitive elements in the scaffolds longer than 1,000 bp in each genome assembly were identified by RepeatMasker (version open-4.0.0 [70]) run with rmblastn (version 2.2.23+) against the arthropod repeats within RepBase Update (20120418) [71]. After these were masked in the assemblies, novel repetitive elements were identified by RepeatScout (version 1.0.5) [82], and those appearing at least 10 times in the genome were counted by RepeatMasker with assembly-specific repeat libraries. To access whether the novel repetitive elements identified in MPB could also be found in *T. castaneum*, we used RepeatMasker with the MPB assembly-specific repeat libraries after the *T. castaneum *genome was similarily masked with arthropod repeats within RepBase Update.

### Analysis of horizontal gene transfer

To confirm the presence of sucrose-6-phosphate hydrolase (*scrB*) in the genome of MPB, we used a reverse primer in this gene and a forward primer in the adjacent hypothetical gene (Table 3) to amplify contiguous portions of these genes and the intervening intergenic region from genomic DNA. To determine whether this *scrB *is present in other *Dendroctonus *species, we used internal primers (Table 3) based upon the DNA and mRNA sequences of *scrB *from MPB and the mRNA sequence from SPB to amplify a segment approximately 1,100 bp in length with DNA from MPB, *D. micans*, and *D. punctatus*. The amplicons were cloned into pJET1.2 (Fermentas) and fully sequenced.

**Table 3:** Primer sequences

Name	Direction	Sequence
*hypo*	Forward	GGTGCTGCCTTTTCTTTGCTATTT
*scrB*	Reverse	CCCAATAACCACATACCAAGACC
*scrB *(internal)	Forward	CCAACATGGCTGGATGAATGACCC
	Reverse	CCTGAGCGCCTCCGTCTCTTTC

### Genome-wide SNP analyses

To examine the variation and distribution of SNPs across the genome, we mapped short-read sequences of genomic DNA from pooled beetles (nine to fourteen beetles per pool) sampled from seven locations in Canada (three in BC; four in AB) and one location from USA (SD) (see Additional file 4, Table 2) to the draft genome assembly, and then identified SNPs. The sequences from all populations were mapped as one dataset to the male assembly using CLC Genomics Workbench (version 5.0.1) using the following parameters: similarity 0.9; length fraction 0.5; insertion, deletion, and mismatch cost 3; min/max paired distance 200/600. The SNPs were then detected using the following parameters: window length 51, minimum quality 20, minimum coverage 20, required variant count 3, minimum variant frequency 6.25%.

### Interspecies comparison

To compare the MPB genome with other sequenced insect genomes for orthologous groups and potential gene-family expansions, we obtained protein sequences for *B. mori, A. pisum, A. mellifera, D. melanogaster, and T. castaneum *from the OrthoMCL-DB [83] and NCBI genomes FTP site, and compared them with the gene models of MPB using OrthoMCL [84] using default parameters and an e-value of less than 1 × 10^-10^.

### Manual annotation of specific gene families

Using reciprocal BLAST against NCBI nr and gene family-specific datasets, the gene families of cytochromes P450, GSTs, and PCWDEs were identified in the genome assembly. Each gene model was manually annotated, and then the non-redundant translated proteins were aligned with MUSCLE [85] to the corresponding proteins from several other insect species for which genomes have been sequenced. A maximum-likelihood phylogeny was created with FastTree 2 [86], and drawn with iTOL [87].

### Data access

These Whole Genome Shotgun projects have been deposited at DDBJ/EMBL/GenBank under accession numbers [APGK00000000] (male) and [APGL00000000] (female). The versions described in this paper are the first versions, [APGK01000000] and [APGL01000000]. The raw sequence data have been submitted to NCBI SRA with BioProject ID SRP014975, the assemblies have been submitted to NCBI with BioProject IDs PRJNA162621 (male) and PRJNA179493 (female), and the Tria Project is represented by NCBI umbrella BioProject PRJNA169907.

## Abbreviations

EST: Expressed sequence tag; FTP: File transfer protocol; GST: Glutathione S-transferase; MPB: Mountain pine beetle; MPET: Mate-paired end tag; Mya: Million years ago; NCBI: National Centre for Biotechnology Information; PCWDE: Plant cell wall-degrading enzymes; PET: Paired end tag; RNA-seq: RNA sequencing; *scrB*: Sucrose-6-phosphate hydrolase; SNP: Single-nucleotide polymorphism; SNV: Single-nucleotide variant.

## Competing interests

The authors declare that they have no competing interests.

## Authors' contributions

CIK, SJMJ, and JB conceived of the study. CIK, ML, and HH prepared genomic DNA, and investigated the horizontally transferred gene by PCR and sequencing. YZ, PP, and RM prepared the sequencing libraries and directed the sequencing. NYL, TRD, SKC, GAT, DLP, SDJ, IB, and SJMJ refined ABySS, and subsequently assembled and annotated the genome. CIK, MMSY, and AN completed the analyses for shared synteny and the sex chromosomes. CIK, MMSY, and DPWH manually annotated and analyzed the described gene families. CIK, MMSY, JKJ, and FAHS completed the SNP analyses. CIK and JB wrote the manuscript. All authors read and approved the final manuscript.

## Supplementary Material

Additional file 1 **Supplementary **Figure 1 Schematic of origin of neo-X and neo-Y in mountain pine beetle (MPB). Click here for file

Additional file 3 **Supplementary **Table 1 Repeat analyses. Click here for file

Additional file 2 **Supplementary **Figure 2 Schematic of (A) simple bubbles and (B) complex bubbles. Click here for file

Additional file 4 **Supplementary **Table 2 Localities of mountain pine beetle (MPB) samples used for genomic DNA sequencing for single-nucleotide polymorphism (SNP) analysis. Click here for file
