# Supplementary material for: Draft genome of the mountain pine beetle, Dendroctonus ponderosae Hopkins, a major forest pest
Source: Genome Biol. 2013 Mar 27;14(3):R27. doi: 10.1186/gb-2013-14-3-r27 (PMC4053930; doi:10.1186/gb-2013-14-3-r27)

Supplementary Figure 1: Schematic of origin of neo-X and neo-Y in MPB.

The male MPB karyotype of 11 AA + neo-XY is thought to have originated from an ancestral state of 12 AA + Xy_p by a fusion of the ancestral X with the largest autosome (in blue) to become neo-X, followed by a loss of the ancestral y_p, and the homozygous daughter chromosome of the fused autosome becoming neo-Y [31, 32]. Thus, large portions of neo-X and neo-Y will have shared synteny originating from the ancestral autosome but may be more divergent than homozygous autosomal pairs.

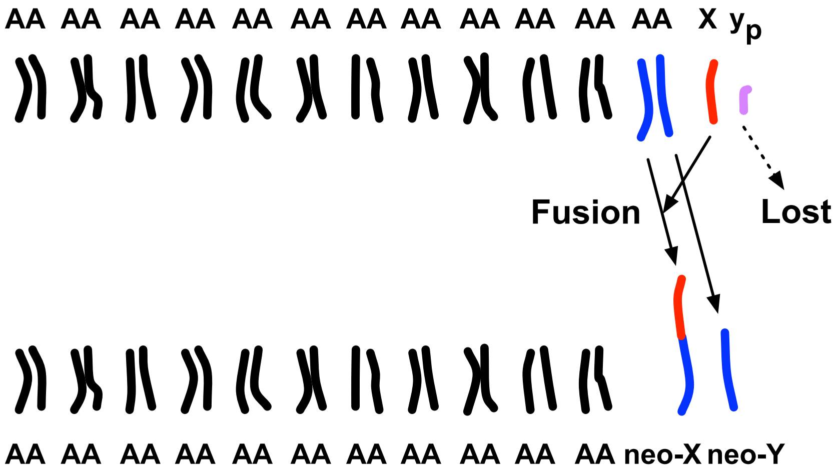

Supplement: Additional file 1 — Supplementary Figure 1 Schematic of origin of neo-X and neo-Y in mountain pine beetle (MPB). [file gb-2013-14-3-r27-S1.PDF]
