# Supplementary material for: Draft genome of the mountain pine beetle, Dendroctonus ponderosae Hopkins, a major forest pest
Source: Genome Biol. 2013 Mar 27;14(3):R27. doi: 10.1186/gb-2013-14-3-r27 (PMC4053930; doi:10.1186/gb-2013-14-3-r27)
Supplement: Additional file 3 — Supplementary Table 1 Repeat analyses. [file gb-2013-14-3-r27-S3.PDF]

Supplementary Table 1 – Repeat analyses

Assembly	Male			Female		
**Number of scaffolds >1000 bp:**	8468			6557		
**Total length (bp)**	255457663 (204355029 excluding N's)			262614799 (214059021 excluding N's)		
**GC content (%)**	36.00			36.25		
	**Number of elements**	**Length occupied (bp)**	**Portion of total length (%)**	**Number of elements**	**Length occupied (bp)**	**Portion of total length (%)**
**Retroelements**	4707	992903	0.39	5348	1160560	0.44
**SINEs:**	16	1068	0	29	1866	0
**Penelope**	116	12258	0	348	51206	0.02
**LINEs:**	2930	564577	0.22	3456	665639	0.25
**CRE/SLACS**	0	0	0	1	51	0
**L2/CR1/Rex**	746	154782	0.06	798	155464	0.06
**R1/LOA/Jockey**	1552	306630	0.12	1791	368366	0.14
**R2/R4/NeSL**	24	3431	0	25	2789	0
**RTE/Bov-B**	450	85152	0.03	449	85047	0.03
**L1/CIN4**	19	954	0	20	982	0
**LTR elements:**	1761	427258	0.17	1863	493055	0.19
**BEL/Pao**	591	139951	0.05	556	149208	0.06
**Ty1/Copia**	227	59908	0.02	272	79359	0.03
**Gypsy/DIRS1**	943	227399	0.09	1035	264488	0.1
**Retroviral**	0	0	0	0	0	0
**DNA transposons**	3804	782130	0.31	4172	822620	0.31
**hobo-Activator**	499	61671	0.02	488	60918	0.02
**Tc1-IS630-Pogo**	1693	525329	0.21	1912	556850	0.21
**En-Spm**	0	0	0	0	0	0
**MuDR-IS905**	3	1978	0	5	2840	0
**PiggyBac**	12	1918	0	12	1713	0
**Tourist/Harbinger**	20	4831	0	14	2777	0
**Other (Mirage, P-element, Transib)**	32	3917	0	25	3180	0
**Rolling-circles**	0	0	0	0	0	0
**Unclassified:**	486	40921	0.02	586	54139	0.02
**Total interspersed repeats:**		1815954	0.71		2037319	0.78
**Small RNA:**	63	6057	0	123	18664	0.01
**Satellites:**	4	334	0	3	141	0
**Simple repeats:**	25266	1033239	0.4	24612	994824	0.38
**Low complexity:**	5683	263655	0.1	5691	262831	0.1
**RepeatScout (unclassified)**	280077	40854370	15.99	292889	57751499	21.99
**Total**		**43959063**	**17.21**		**61050581**	**23.25**
