# Supplementary figures and images for: Draft genome of the mountain pine beetle, Dendroctonus ponderosae Hopkins, a major forest pest
Source: Genome Biol. 2013 Mar 27;14(3):R27. doi: 10.1186/gb-2013-14-3-r27 (PMC4053930; doi:10.1186/gb-2013-14-3-r27)

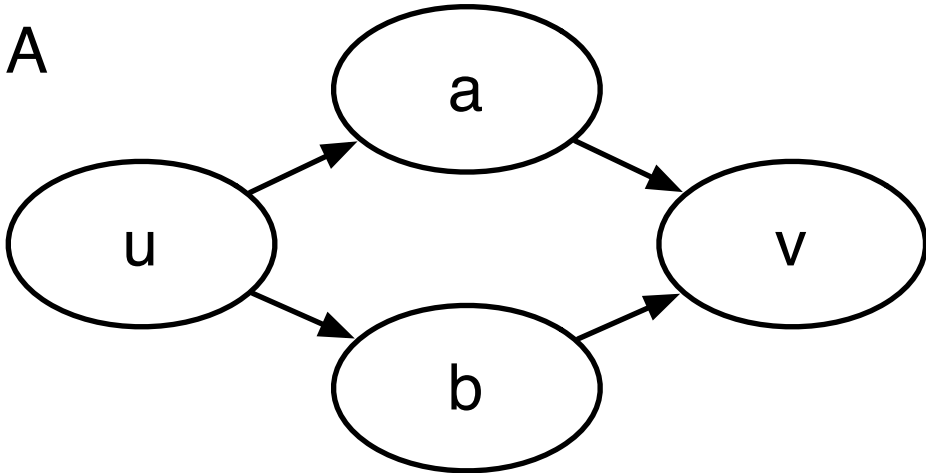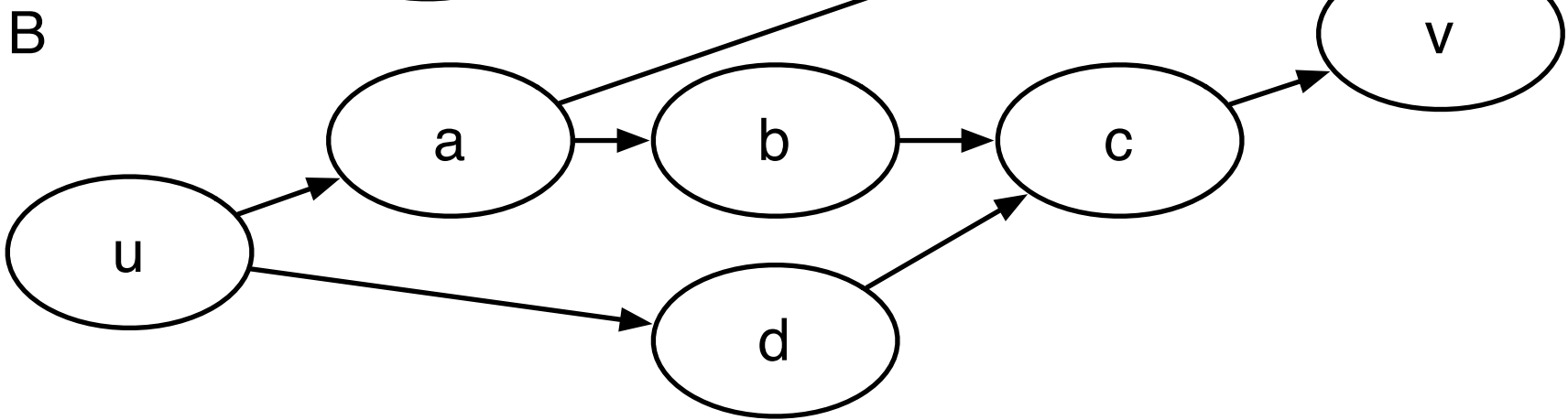

Supplement: Additional file 2 — Supplementary Figure 2 Schematic of (A) simple bubbles and (B) complex bubbles. [file gb-2013-14-3-r27-S2.PDF]
