# Supplementary material for: Draft genome of the mountain pine beetle, Dendroctonus ponderosae Hopkins, a major forest pest
Source: Genome Biol. 2013 Mar 27;14(3):R27. doi: 10.1186/gb-2013-14-3-r27 (PMC4053930; doi:10.1186/gb-2013-14-3-r27)
Supplement: Additional file 4 — Supplementary Table 2 Localities of mountain pine beetle (MPB) samples used for genomic DNA sequencing for single-nucleotide polymorphism (SNP) analysis. [file gb-2013-14-3-r27-S4.PDF]

Supplementary Table 2 - Localities of MPB samples used for genomic DNA sequencing for SNP analysis

Location	Site	Latitude	Longitude	# individuals pooled	NCBI SRA accession
**Alberta, Canada**					
	Cypress Hills	49.57617	-109.97483	14	SRX035175
	Fairview	56.12840	-118.54750	10	SRX031642
	Kananaskis	51.01944	-115.14839	10	SRX035174
	Whitecourt	54.13600	-116.03150	13	SRX031643
**British Columbia, Canada**					
	Houston	53.99397	-126.65272	9	SRX035177
	Terrace	54.45660	-128.51530	10	SRX031641
	Valhalla	49.75033	-117.51808	11	SRX035176
**South Dakota, USA**					
	Black Hills	43.96120	-103.57910	10	SRX031095
